# Bioinspired total syntheses of natural products: a personal adventure

**DOI:** 10.3762/bjoc.21.160

**Published:** 2025-10-09

**Authors:** Zhengyi Qin, Yuting Yang, Nuran Yan, Xinyu Liang, Zhiyu Zhang, Yaxuan Duan, Huilin Li, Xuegong She

**Affiliations:** 1 State Key Laboratory of Natural Product Chemistry, College of Chemistry and Chemical Engineering, Lanzhou University, Lanzhou 730000, P. R. China; 2 State Key Laboratory of Green Pesticide, Guizhou University, Guiyang 550025, P. R. China

**Keywords:** bioinspired total synthesis, chabranol, gymnothelignans, monocerin, sarglamides, taberginggine

## Abstract

Bioinspired total synthesis represents an important concept to guide the designing of powerful synthetic strategies. Our group has a long-time interest and experience in designing synthetic strategies through analyzing the biosynthetic pathway of natural products. Recently, we have achieved an array of bioinspired total syntheses, which showed the great power of this approach in natural product synthesis. Documented herein is a review of these achievements which include the detailed process of how we develop these strategies. Specifically, bioinspired total synthesis of three types of natural products, namely diterpenoids (chabranol, and monocerin), alkaloids (indole, hydroquinoline, and monoterpenoid−indolidinoid hybrid), and gymnothelignans are discussed. Based on these achievements on bioinspired total synthesis, we provide some information on how to use this important strategy in natural product synthesis.

## Introduction

Natural products are chemical substances generated within living organisms in nature. They are products of biotic evolution in which live survives from changes of Earth environment by changing themselves. Natural products play a pivotal role in biological transformations within organisms, and moreover, are of great value for human life by serving as food, cloth and medicine. It represents a longstanding goal in human history to obtain natural products and develop new applications. Traditionally, natural products are directly obtained from its natural source, such as sugar and vitamins. Since Wöhler’s historic success [1] in converting widely believed “inorganic” materials into the “organic” compound urea, people began to be aware of the capability of mankind in making natural organic molecules. Since then, organic scientists are brave to challenge the complex organic structure of natural products given by nature [2–5]. To achieve the growing structural complexity of natural products, the synthetic capability have been increasing all the time by discovering and inventing a vast number of new organic reactions and methodologies [6]. However, could mankind become really stronger than Mother Nature one day? This question seems to have no answer as future is unpredictable. Maybe, Mother Nature could be our teacher and elevate our synthetic capability in some way.

**Figure 1:**
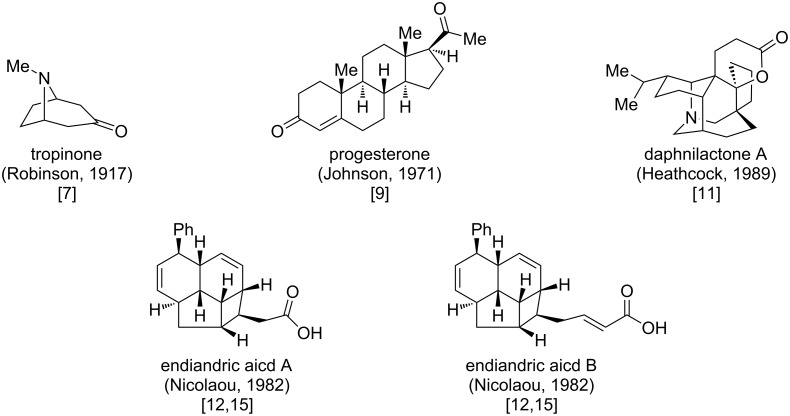
Representative natural products with biomimetic total synthesis.

In academia, learning from nature from the synthetic point of view has a very long history. A remarkable example is Robinson’s tropinone synthesis early in 1917 [7] (Figure 1). This historic event may be ahead of its time and it allows rapid assembly of a complex natural product with a three-dimensional framework in a cascade way. Later, this was regarded as an artificial mimic in laboratory of the biochemical transformations in nature. In the 20th century, Woodward elevated the field of natural product total synthesis to the artistic status [8], and Corey drove it to a precise science full of chemical logics [6]. In this period, a lot of biomimetic total synthesis came out, such as Johnson’s progesterone synthesis [9–10] Heathcock’s synthesis of daphniphyllum alkaloids [11] and Nicolaou’s synthesis of endiandric acids [12–15] (Figure 1). The bioinspired total synthesis literally showed the great power to gain complexity. In the 21st century, biomimetic or bioinspired synthesis has been widely realized as a powerful concept approach to natural product total synthesis, and a lot of total synthetic works of this kind have been reported.

A bioinspired approach represents many advantages to bring benefits to total synthesis. It could rapidly achieve complexity of the target molecule from a much simpler precursor in diverse forms of transformations such as cascade reaction, cycloaddition, and C–H functionalization, thereby, shorten the synthetic steps and gain efficiency. More significantly, since the exact biosynthetic pathway of a natural product is generally very complicated and hard to elucidate clearly, a biosynthetic pathway is basically proposed by the isolating scientist according to the structural analysis of the symbiotic natural products. The proposal lacks strong evidences, no matter it is scientifically reasonable or not. The bioinspired synthetic would provide evidences to support such a plausible biogenetic pathway through chemical transformations under simple biomimetic reaction conditions like acid, base, or visible light.

How to design a bioinspired approach may be most attractive to synthetic chemists. Recently, Tang [16] and Jia [17] independently reviewed their remarkable bioinspired total syntheses as accounts. Tang documented their longtime carrier of learning from nature aiming to achieve better results than nature. Jia categorized their works into three sections to showcase how they learn from nature, including 1) to mimic the key cyclization steps, 2) to mimic the revised biosynthetic pathway proposed, and 3) to mimic the skeletal diversification process. These three types of bioinspired synthesis probably lead this field to the lane of scientific logic, which would provide guidelines to design a bioinspired strategy.

Our group has a long-time of research experience on complex natural product synthesis. To achieve higher efficiency of synthesis, we inevitably exploited the concept of biomimetic or bioinspired total synthesis. Indeed, this approach has been proved to be of great power to access molecular complexity, and it is applicable to diverse types of natural products such as terpenoids, alkaloids, polyketides and lignans. Herein, we document our adventure of bioinspired total synthesis of natural products.

## Review

### Synthesis of chabranol

In 2009, Duh and co-workers investigated the ingredients of Formosan soft corals *Sinularia capillosa* Tixier-Durivault and *Nephthea chabroli* Audouin, collected from west pacific Dongsha Atoll and Siaoliouciou Island, providing two terpenoid natural products capillosanol and chabranol, respectively [18]. Chabranol was identified to contain a new bridged skeleton through extensive NMR experiments. However, no single-crystal X-ray diffraction analysis was conducted, making the structural determinations not that solid. It showed moderate cytotoxicity against P-388 (mouse lymphocytic leukemia). Attracted by the novel bridged structure and in order to further determine the structure, particularly the absolute configurations, we explored the total synthesis of chabranol [19].

**Scheme 1:**
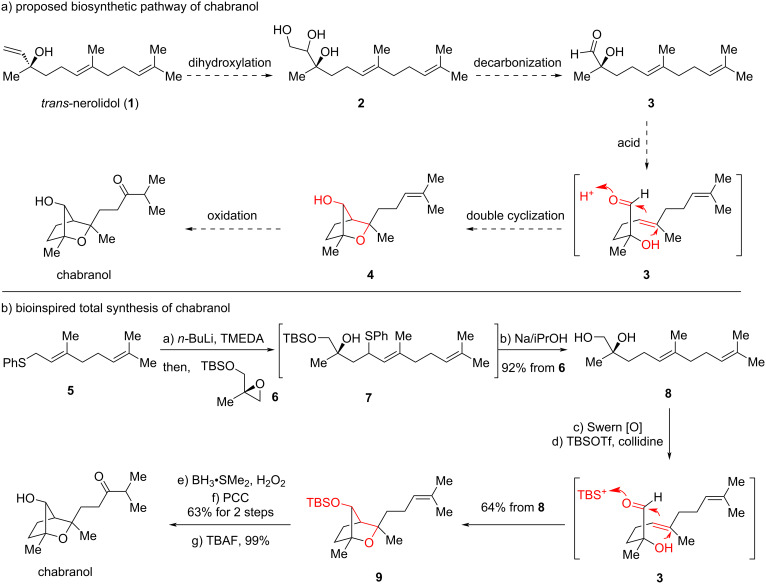
Bioinspired total synthesis of chabranol (2010).

Structurally, this molecule contains an oxa-[2.2.1] bridge, with two quaternary centers including one at the bridgehead position. To establish a proper strategy for total synthesis, we could, somehow, design its retrosynthetic analysis through diverse approaches to construct such a bicyclic skeleton. However, inspired by the biomimetic polycyclization of terpenoids, we sought to propose the biosynthetic pathway, which has not yet been reported in Duh’s isolation report (Scheme 1a). In our proposal, the linear sesquiterpenoid *trans*-nerolidol (**1**) with a chiral tertiary alcohol undergoes dihydroxylation to generate triol **2**, which further proceeds a C–C bond cleavage to afford aldehyde **3**. This linear aldehyde would be activated by an acid to trigger a key Prins cyclization with the trisubstituted olefin through reaction model **3** and generate a putative tertiary carbocation to be trapped by the chiral alcohol, providing bicycle **4** stereoselectively. Finally, the last olefin would be oxidized to ketone, which gives chabranol.

According to this biosynthetic proposal, we thought that the Prins-triggered double cyclization would serve as a powerful method to construct the bicycle in one step, and moreover, the proposed Prins-based double cyclization needs further supporting evidences of chemical transformations. Thus, a bioinspired total synthesis was investigated (Scheme 1b). Synthetically, we did not start from *trans*-nerolidol (**1**) to construct a C–C bond cleavage. Instead, a convergent coupling approach was selected to quickly access the aldehyde precursor. Phenyl sulfide **5**, derived from phenylthiol and geranyl bromide, coupled with chiral epoxide **6**, prepared through Sharpless epoxidation and TBS protection of 2-methylprop-2-en-1-ol, under strong basic conditions to generate intermediate **7** to further reduce the sulfide moiety with sodium, furnishing diol **8** with the loss of TBS protection in one pot. Oxidation of the primary alcohol using Swern oxidation gave the hydroxy aldehyde **3**, which was activated with a formal silicon cation to trigger the Prins cyclization terminated by the tertiary alcohol, affording silylated bicycle **9** directly through the designed bioinspired approach. This key reaction mimics the plausible biosynthetic pathway and demonstrates great efficiency and sole diastereoselectivity, showcasing the plausibility of the biosynthetic proposal. Further redox manipulations of the last olefin and deprotection ultimately provided chabranol. To clearly confirm the structure further, X-ray diffraction analysis of the derivative of bicycle **9** was obtained. This approach established the first total synthesis of chabranol in a concise way through the bioinspired Prins-triggered double cyclization strategy to rapidly construct the bicycle.

### Total syntheses of natural products of the monocerin-family

**Scheme 2:**
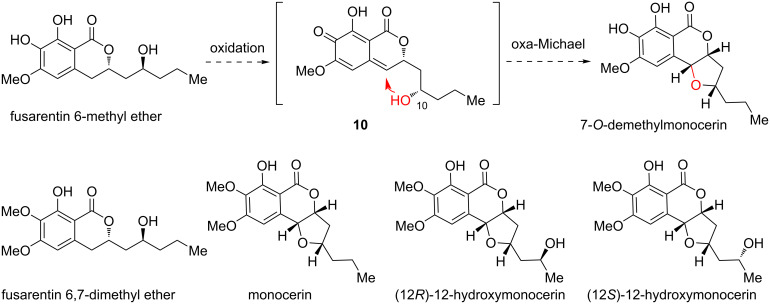
Proposed biosynthetic pathway of monocerin-family natural products.

Early in 1979, monocerin and 7-O-demethylmonocerin were elucidated from *Fusarium larvarum* [20]. Since then, a large group of analogues have been isolated from diverse fungal species [21–25] (Scheme 2). Along with the structural elucidation, biological studies of these molecules indicated that they exhibit a broad-spectrum activities including antifungal, insecticidal, plant pathogenic properties and phytotoxic activity. Structurally, these molecules basically contain an isocoumarin ring system and a five-carbon side chain. The side chain could further form a *cis*-substituted tetrahydrofuran (THF) moiety fused to the lactone with higher oxidation states. Notably, the phenyl ring contains three oxygen substituents in the form of alcohol and methoxy groups at different positions.

Biosynthetically, the THF ring was supposed to be formed through a benzylic oxidation to generate a *para*-quinone methide (*p*QM) intermediate. Using fusarentin 6-methyl ether as an example, *p*QM intermediate **10** would be generated. The C10 alcohol should successively undergo an oxa-Michael addition reaction to close the THF ring, providing 7-*O*-demethylmonocerin. Similarly, monocerin and 12-hydroxymonocerin were presumably generated from their corresponding precursors through similar oxidation and oxa-Michael addition reactions. Given the fact that quinone methides served as a powerful platform for the development of rich useful organic transformations, especially, through catalytic asymmetric methods [26], we intended to probe this biomimetic oxidative cyclization transformation [27–28].

**Scheme 3:**
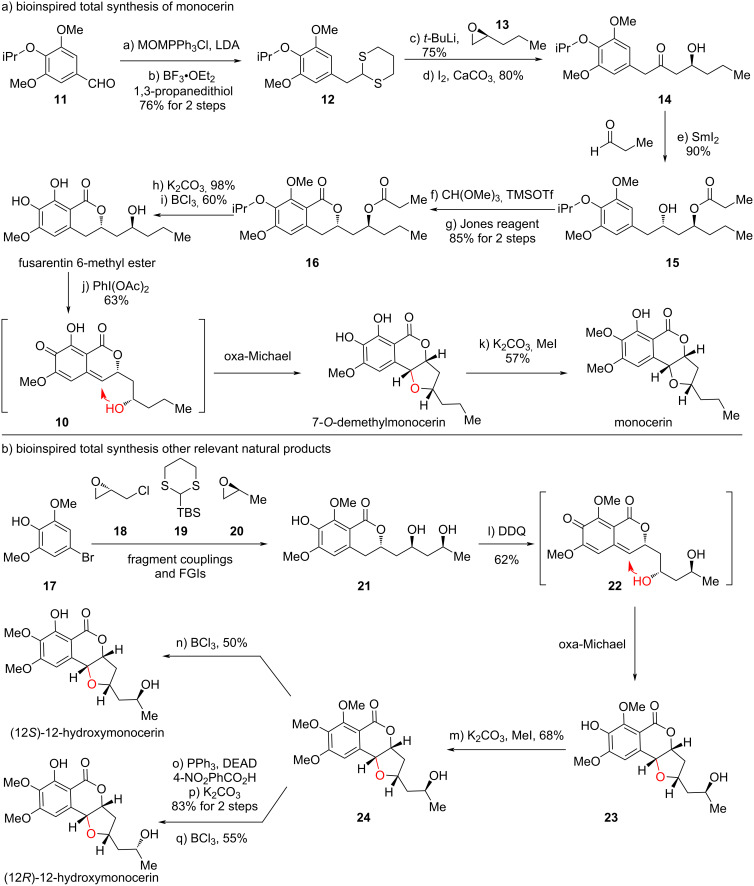
Bioinspired total synthesis of monocerin-family molecules (2013).

In 2013, we first used monocerin as a model target molecule to initiate our study (Scheme 3a). Starting from benzaldehyde **11** with an isopropyl group on the hydroxy group in 4-position, Wittig reaction with MOMPPh_3_Cl and LDA gave the putative methyl enol ether, which could be directly converted into 1,3-dithiane **12** with propane-1,3-dithiol. Nucleophilic addition to chiral epoxide **13** and oxidative hydrolysis of 1,3-dithiane to ketone delivered chiral β-hydroxyketone **14**. Evans−Tishchenko reduction of ketone using SmI_2_ and propionaldehyde provided **15** diastereoselectively. Friedel–Crafts cyclization using trimethyl orthoformate and TMSOTf provided the cyclic acetal moiety to be oxidized with PDC, delivering the isocoumarin skeleton in **16**. Chemoselective removal of the ester with the lactone unreacted, which released the secondary alcohol, followed by BCl_3_-promoted selective cleavage of the isopropyl and one methyl protection ultimately furnished thenatural product fusarentin 6-methyl ether.

With fusarentin 6-methyl ether in hand, we explored the bioinspired oxidation/oxa-Michael addition. This transformation was successfully achieved by using PhI(OAc)_2_ as oxidant, providing 7-*O*-demethylmonocerin, containing the *cis*-substituted THF ring with sole diastereoselectivity. Site-selective mono-methylation gave rise to monocerin.

The bioinspired approach successfully found applications in the total synthesis of the monocerin-type natural products bearing a C10 alcohol. However, for other molecules with a C12 alcohol, it is unknown whether the C12 alcohol would bring challenges to the oxa-Michael addition or not. To investigate this, we used fragments **17**–**20** to access diol substrate **21**, through Smith’s 1,3-dithiane linchpin coupling (Scheme 3b). To our delight, using DDQ as the optimal oxidizing reagent, the expected THF-containing product **23** was generated as a sole diastereoisomer, suggesting the *p*QM **22** underwent oxa-Michael addition only with the C10 alcohol. The free C12 alcohol did not affect this process, which further supported the probability of the proposed biosynthetic approach. Subsequently, site-selective methylation on phenol provided **24**, which further underwent *O*-directed demethylation to afford (12*S*)-hydroxymonocerin. On the other hand, a two-step protocol involving Mitsunobu reaction with an acid and base-promoted saponification inversed the C12 alcohol stereochemistry, which ultimately provided (12*R*)-hydroxymonocerin.

### Total synthesis and bioinspired skeletal diversification of (12-MeO)-tabertinggine

In 2013, Kam and co-workers reported the discovery of two novel indole alkaloids voatinggine and tabertinggine from the plants of *Tabernaemontana* (Apocynaceae) genus in Malayan, which are literally widely distributed in tropical America, Africa, and Asia [29]. Given the fact that these plants are rich sources of bioactive alkaloids, voatinggine and tabertinggine with unprecedented skeletons are of great interests to synthetic chemists, since the biological studies are limited due to material scarcity in the nature.

**Scheme 4:**
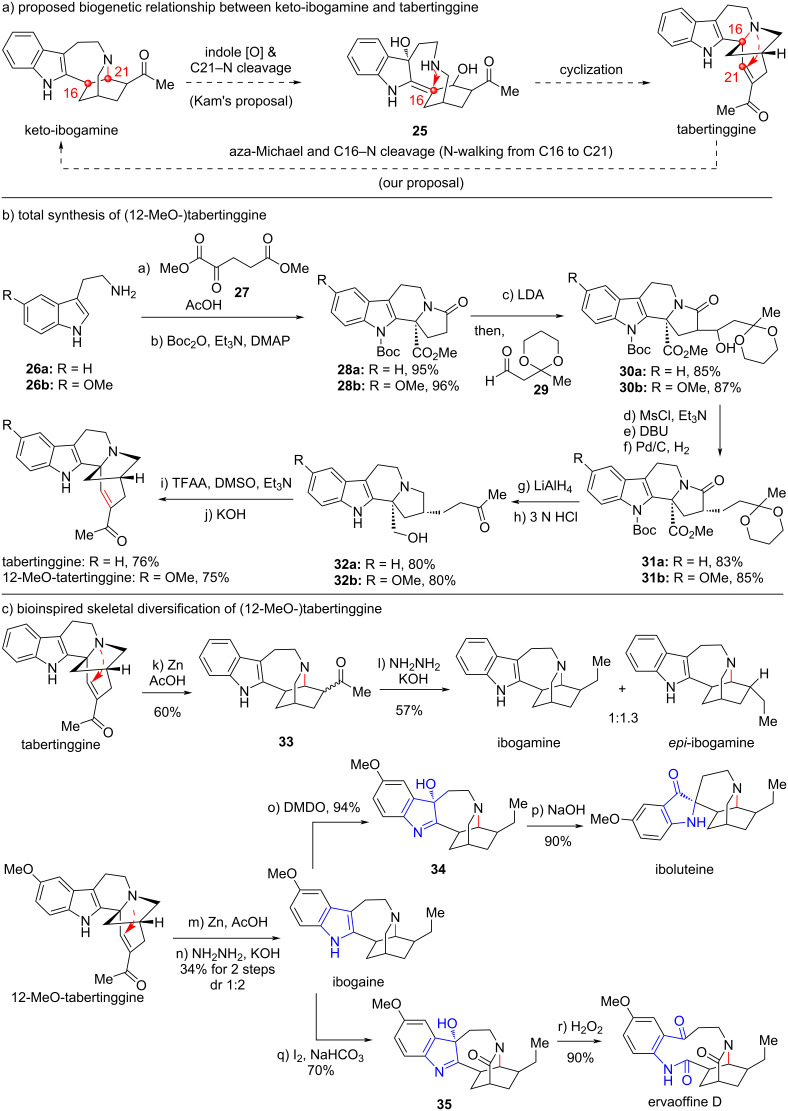
Bioinspired skeletal diversification of (12-MeO-)tabertinggine (2016).

Structurally, tabertinggine contains an aza-[3.2.1] bridged skeleton linked to indole C2 position. To rationalize how this unique structure is generated in nature (Scheme 4a), Kam proposed that tabertinggine might be biosynthetically generated from an ibogamine precursor keto-ibogamine through an indole oxidation and C21–N bond cleavage process to give intermediate **25**, which further undergoes a cyclization to form the C16–N bond and dehydration to generate the enone. By analyzing the structure, we supposed that there might exist an inversed pathway in which tabertinggine could be converted into the ibogamine aza-[2.2.2] bridged skeleton through intramolecular aza-Michael addition of the enone moiety to form the C21–N bond and a subsequent C16–N bond cleavage process (N-walking from C16 to C21) [30]. To investigate this proposal, we first developed the total synthesis of tabertinggine to obtain enough material for biogenetic skeletal diversification (Scheme 4b).

As shown in Scheme 4b, the total synthesis of tabertinggine started from the Pictet–Spengler cyclization of tryptamine **26a** with keto-diester intermediate **27** followed with a lactamization reaction in one-pot to access the γ-lactam moiety in product **28a**, which was obtained after Boc protection. Base-promoted aldol reaction of lactam **28a** with aldehyde **29** gave rise to alcohol **30a**, which subsequently underwent dehydroxylation protocol involving base-promoted mesylate elimination and catalytic hydrogenation reactions, providing **31a**. Reduction of lactam and ester in one pot with LiAlH_4_ and acid-promoted hydrolysis of ketal protection to ketone furnished **32a**. Finally, oxidation of the primary alcohol to aldehyde followed by base-promoted aldol condensation reaction successfully provided tabertinggine. This approach achieved the first total synthesis of tabertinggine in only ten steps and capable of supplying enough material for the following skeletal diversifications.

With sufficient tabertinggine in hand, we treated it to Zn powder in AcOH to facilitate the N-walking transformation, and generated keto-ibogamine product **33** as a mixture of diastereomers (Scheme 4c). Then, reduction of ketone to methylene under Wolff–Kishner–Huang conditions provided ibogamine and its epimer in a 1:1.3 ratio. This result supported our proposal that tabertinggine might be the biogenetic precursor of ibogamine.

To further access some other natural products through skeletal diversifications, we found that some target molecules contain an OMe group at C12 position. So, 12-OMe-tabertinggine was prepared from C5-OMe-tryptamine **26b** just following the same route for synthesis of tatertinggine, and the yield of each step was basically comparable (Scheme 4b). Starting from 12-OMe-tabertinggine, Zn-promoted N-walking and ketone reduction provided ibogaine in 1:3 ratio with the major epimer. Indole oxidation with DMDO provided **34** as a single isomer, which further underwent a migratory rearrangement and afforded iboluteine. On the other hand, oxidation of ibogaine with molecular iodine achieved both indole and amine oxidations, delivering lactam **35**. Intermediate **35** could be oxidized with H_2_O_2_ through C–C bond cleavage to give natural product ervaoffine D. Thus, through the bioinspired skeletal diversifications of (12-OMe)-tabertinggine, four iboga alkaloids could be powerfully synthesized.

### Bioinispired total synthesis of gymnothelignans

**Scheme 5:**
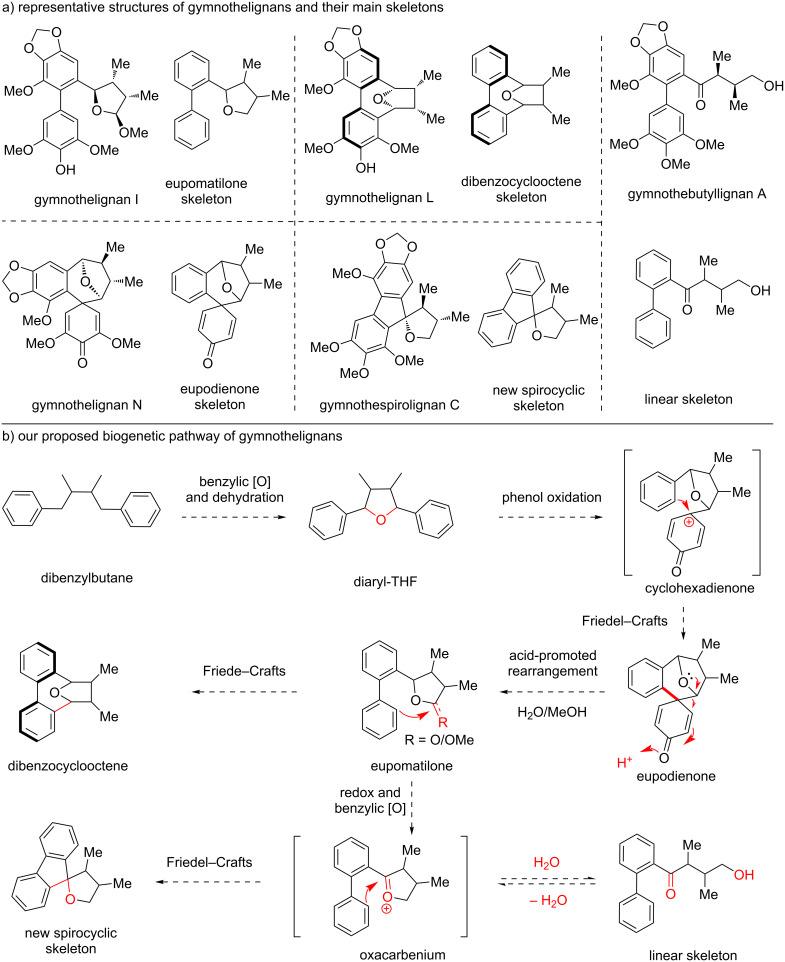
Structures and our proposed biosynthetic pathway of gymnothelignans.

The *Gymnotheca* (Saururaceae) genus only contains two species of plants, *Gymnotheca chinensis* Decne and *Gymnotheca involucrate* Pei. These two plants are endemic in China, and have been used as medicinal herb for a long history to treat diseases such as dysentery, abdominal distention, edema, contusion, and strains [31]. Since 2012, Zhou and co-workers extensively investigated the ingredients of these plants, and have elucidated a huge number of lignan natural products with novel structural backbones [32–35] (Scheme 5a). Biologically, the gymnothelignans exhibit a broad-spectrum of properties such as antiviral, antifungal, and insecticidal activities.

Structurally, gymnothelignans included five types of common skeletons, namely eupomatilone, eupodienone, dibenzocyclooctene, and a novel spirocyclic and a linear skeleton (Scheme 5a). Notably, each of the skeleton comprises a series of members with diverse forms of oxidation states or dimers, and only one member is displayed. It is also worth noting that the former three types of lignans were isolated first from *Gymnotheca chinensis* Decne and the spirocyclic and linear members were latterly discovered from *Gymnotheca involucrate* Pei.

With respect to the biogenetic pathway of gymnothelignans, Zhou tentatively proposed a biosynthetic approach for the early isolated members of eupomatilone, eupodienone, and dibenzocyclooctene skeletons. We analyzed the structures and proposed a new biogenetic pathway of gymnothelignans with some differences to Zhou’s proposal [36–39]. As shown in Scheme 5b, the dibenzylbutane skeleton might undergo benzylic oxidations and intramolecular dehydration to access a diaryltetrahydrofuran (THF) skeleton. Then, oxidation of one aryl group through phenol oxidation would generate a putative cyclohexadienone intermediate to be further captured by the other phenol to access eupodienone through a Friedel–Crafts reaction. Next, the cyclohexadienone moiety could be activated by an acid to undergo a rearrangement reaction to provide eupomatilone. The exocyclic THF ring might exist as diverse forms such as hemiacetal, acetal, lactone or acetal-linked dimer. The (hemi)acetal moiety could generate an oxa-carbenium cation triggered by an acid to proceed a Friedel–Crafts reaction to afford the dibenzocyclooctene skeleton. On the other hand, eupomatilone would undergo redox transformations to generate another oxa-carbenium cation to undergo a Friedel–Crafts reaction to form a spirocyclic skeleton. This oxa-carbenium cation could also undergo hydrolysis to provide a linear skeleton with a hydroxy ketone moiety, and this hydrolysis process is reversible. It is worth noting that Zhou explored the chemical conversion of the eupodienone skeleton to the eupomatilone skeleton through acid-promoted rearrangement under the conditions of H_2_SO_4_/MeOH, since their isolation of the natural products [32]. However, other critical bioinspired transformations have not yet been explored, which prompted us to initiate this study.

**Scheme 6:**
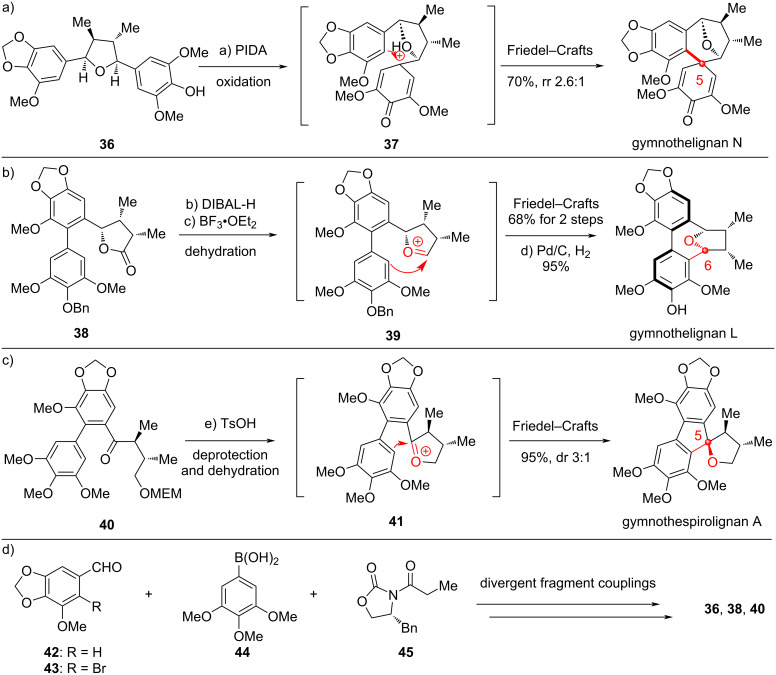
Bioinspired total synthesis of gymnothelignans (2014–2025).

Guided by this biogenetic proposal, we conducted a series of reactions to probe the proposed key transformations (Scheme 6). Starting from the diaryl-THF-type precursor **36** with a phenol moiety, oxidation of the phenol with hypervalent iodine reagent PIDA generated the putative oxa-carbenium intermediate **37**, which successfully underwent the Friedel–Crafts cyclization to provide gymnothelignan N site-selectively [36] (Scheme 6a). The favored position of the Friedel–Crafts cyclization is literally the expected one, and it matches with the case in nature for generation of the natural product gymnothelignan N. This reaction successfully converts an open diaryl-THF-type precursor into an eupodienone skeleton. Our screened reaction conditions for the oxidative Friedel–Crafts cyclization were re-conducted by Lee and co-workers to show similar results for the synthesis of gymnothelignan N [40]. Then, we prepared the eupomatilone precursor **38** with a diphenyl and a lactone moiety through a stereoselective de novo approach [37]. The lactone was reduced with DIBAL-H to a semiketal, which was treated with the Lewis acid BF_3_·OEt_2_ to generate the putative oxa-carbenium **39**. This cation triggered the Friedel–Crafts proposed cyclization with the electron-rich phenyl ring to give the dibenzocyclooctene product, which finally afforded the corresponding natural product gymnothelignan L after removal of the Bn protection (Scheme 6b). Interestingly, this transformation not only provided the expected skeleton, but also showed sole stereoselectivity in constructing the axially chiral diphenyl moiety. The obtained axial chirality is identical to the naturally occurring one. Almost at the same time, Soorukram and co-workers reported the same approach to access the dibenzocyclooctene member gymnothelignan V [41]. Next, we examined the bioinspired transformation of the linear skeleton to the spirocycle. By using chiral compound **40** with an acid-sensitive protecting group MEM on the alcohol as the precursor, the simple Brønsted acid TsOH successfully promoted the deprotection and dehydration to generate oxa-carbenium **41**, which subsequently proceeded the Friedel–Crafts cyclization to furnish gymnothespirolignan A in excellent yield and good diastereoselectivity [38] (Scheme 6c). The minor epimer is gymnothespirolignan C, another natural product of this family. This cascade reaction supports the proposed biogenetic pathway of the newly isolated novel siprocyclic gymnothelignans. The bioinspired double cyclization to forge the spirocycle in one step represents a comparably more powerful method to rapidly generate complexity, since the de novo synthesis approach by Cuny and co-workers [42] has inevitably met with a lot of problems on stereoselectivity although they started the synthesis from a tricyclic fluorenone to mainly focus on the construction of the THF system.

These bioinspired transformations served as solid evidences of chemical conversions to support the plausible biogenetic pathway of how the diverse skeletons of gymnothelignans were generated in nature. Moreover, these studies also provide powerful routes to the asymmetric total synthesis of these bioactive molecules. The precursors of the key bioinspired transformations **36**, **38** and **40** were efficiently synthesized from simple fragments aryl aldehyde **42** or **43**, phenylboronic acid **44** and chiral auxiliary-containing building block **45**, through divergent coupling approaches, and the stereocontrol with Evans oxazolidinone was always reliable to obtain an sole diastereomer (Scheme 6d).

### Bioinspired concise and scalable total synthesis sarglamides

**Scheme 7:**
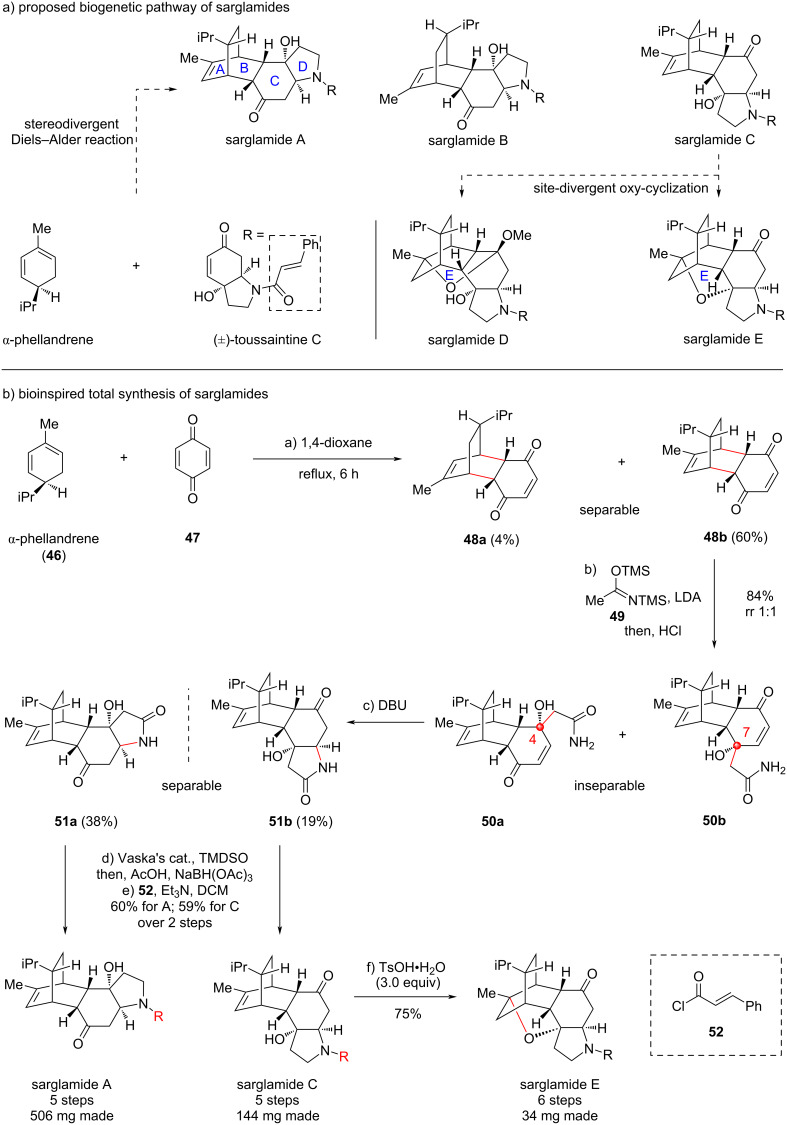
Bioinspired total synthesis of sarglamides (2025).

In 2023, Yue and co-workers investigated the ingredients of the Chinese folk medicine *Sarcandra glabra* subsp. *brachystachys*, and discovered a series of complex natural products named sargalmides A–E [43] (Scheme 7a). To clearly elucidate the complex structures, Yue and co-workers used multiple methods including extensive spectroscopic analysis (NMR, IR and MS), X-ray crystallography, quantum-chemical calculations, and chemical transformations. Biologically, these molecules exhibit antineuroinflammatory activity against lipopolysaccharide (LPS)-induced inflammation in BV-2 microglial cells. Structurally, these molecules generally have a tetracyclic backbone (A–D rings), among which AB rings form a bridged [2.2.2] carbocycle fused to CD rings. For sarglamides D and E, another oxygen-bridged E ring shows up at different positions. Overall, sarglamides A–E exhibit unusual unprecedented skeletons as they contain both monoterpenoid and indolidinoid subunits. Such a hybrid structure is rarely found in natural products.

Attracted by the structural uniqueness and to further confirm the structures, we initiated to explore the synthesis of these molecules. To design a practical retrosynthesis for these complex natural products, we analyzed the plausible biogenetic pathway of sarglamides to obtain some inspirations (Scheme 7a). Biogenetically, sarglamides A–C are generated directly from α-phellandrene and toussaintine C through stereodivergent Diels–Alder reactions, since the indolidinoid natural product toussaintine C is known and symbiotic with sarglamides. More importantly, a solid evidence to support this proposal is that toussaintine C was isolated as a racemic mixture, and sarglamides A and B are derived from one enantiomer of toussaintine C while sarglamides C–E are derived from the other one. The Diels–Alder cycloadditions are basically proceeded through *endo*-selectivity. The isopropyl group in α-phellandrene provided a steric effect to the cycloaddition. Then, sarglamides D and E arose from C through acid-promoted oxy-cyclizations. This biosynthetic approach rapidly constructs the complex structure from simple precursors, thus we intended to achieve the total synthesis of sarglamides through the bioinspired synthetic approach [44].

Initially, we prepared a bicyclic precursor similar with toussaintine C to react with α-phellandrene to mimic the bioinspired Diels–Alder reaction, which, however, showed no reactivity due to the strong steric hindrance. To decrease the steric effect, we finally used simple 1,4-benzoquinone to act as the dienophile, and the expected Diels–Alder reaction proceeded smoothly to provide the *endo* adducts **48a** and **48b**, in which **48b** was the major product dominated by the isopropyl steric effect. Isomers **48a** and **48b** were separable and determined through X-ray diffraction analysis. The major one **48b** was used for further study to install the pyrrolidine system. Thus, nucleophilic addition of an *N,O*-enolate, derived from precursor **49** with LDA, to the ketone functionality in **48b** was implemented to generate a pair of inseparable regioisomers **50a** and **50b**, arose from C4 and C7 additions, respectively. This result suggests that the addition fully went from the upper face and the left-oriented isopropyl showed no steric effect to control the regioselectivity. With free amide **50a** and **50b** in hand, the mixture was treated with DBU to promote aza-Michael addition to afford lactam compounds **51a** and **51b**, which became separable, gratifyingly. Finally, chemoselective reduction of lactam to amine with the more reactive ketone unreacted was successfully achieved. In this transformation, the iridium-catalyzed hydrosilylation of the lactam generated the corresponding *O*-silyl aminal, which was then treated with AcOH to afford the putative iminium cation to be chemoselectively reduced with NaBH_3_CN to ultimately reach the amine functionality. Through this protocol, the amine product was obtained, but it could not be isolated from silica gel for its high polarity. Thus, the extracted crude material of the amine was directly treated with cinnamoyl chloride under basic conditions to install the cinnamoyl side chain, delivering natural products sarglamides A and C independently. Sarglamide C was exposed to an acid to access sarglamides D and E with an oxyl-bridge. When a catalytic amount of TsOH was used, sarglamides D and E could be obtained with a 1:1 ratio, as conducted by Yue [43] and Tong [45] research groups. In our hand, we used an excess amount of TsOH (3.0 equiv), and found that sarglamide E was generated exclusively. Ultimately, a powerful total syntheses of complex polycyclic sarglamides A, C, D and E were established through a concise and protecting group-free approach, which was definitely inspired and utilized by the analysis of the biogenetic pathway of these natural products. Through this approach, a notable amount of natural products were made in one batch. The synthesis supports the proposed biogenetic pathway, and the *endo*-selective stereochemical outcome of the Diels–Alder reaction in the bioinspired synthesis fully matched with the proposed approach in nature.

## Conclusion

The biomimetic or bioinspired total synthesis of natural product has gone a long way from more than one hundred years ago to today. By analyzing the plausible biogenetic pathway of natural products, one could literally obtain practical inspiration to guide the synthetic work. Utilization of this inspiration could dramatically decrease complexity of the target molecules, and ultimately lead to a powerful synthetic approach. By utilizing the concept of bioinspired total synthesis, our group has achieved the total synthesis of chabranol through a Lewis acid-promoted double cyclization, total synthesis of natural products of the monocerin-family through a benzylic oxidation and oxa-Michael addition reaction, total synthesis of (12-OMe-)tabertinggine converted it into other iboga alkaloids through skeletal diversifications, total synthesis of gymnothelignans of diverse skeletons through acid-promoted Friedel–Crafts cyclization, and the total synthesis of sarglamides through an *endo*-selective Diels–Alder reaction. These examples showcase the power of bioinspired strategies in total synthesis of complex natural products, and also provide the working model of how to utilize and develop a bioinspired total synthesis.

For a simple guidance of how to use the bioinspired total synthesis approach, one could go to figure out the symbiotic or structure-related members of the target natural product before the disconnection, and the structural similarities and differences between them would provide inspiration to the retrosynthetic disconnection pathway. Meanwhile, when a natural product or an advanced intermediate relevant to diverse naturally occurring molecules would be rationalized as a common connecting point of all molecules, a convergent strategy for collective total syntheses of natural products would be exploited through skeletal diversification. Meanwhile, symbiotic natural products involving site-, chemo-, and stereoselective patterns in forming the polycycles could be trace back to same precursors or fragments, which would result in bioinspired total syntheses of natural products through controllable and selective chemical transformations. If there is only one single target molecule with an unprecedented skeleton, we can try to realize how the novel structure is generated in nature from known chiral pool molecules, thus a key cascade reaction to rapidly construct the novel framework would be biosynthetically applicable. This approach has found wide applications in total syntheses of newly-isolated terpenes.

There is no doubt that bioinspired total synthesis is currently a powerful and critical approach for complex natural product total synthesis. However, there still exist great challenges in the field, which would stimulate the discovery of new breakthroughs. Chemo- and regioselective employment of functionalities in a complex framework are formidable challenges for mankind since such transformations in nature are precisely induced by enzymes. Bioinspired functionalization of C–H bond in total synthesis is rarely developed and it represents another challenge, despite numerous methodologies have been invented. Moreover, bioinspired total synthesis involving visible light and enzymes are new significant trends in this field, and these techniques have demonstrated great power in achieving unprecedented selectivity and reactivity. Since nature still prevails in rapid generating molecular complexity and achieving selectivity, it is unarguably that the field of bioinspired total synthesis still has a long way to go.

## Data Availability

Data sharing is not applicable as no new data was generated or analyzed in this study.
